# Advances in Nanosensors for Biological and Environmental Analysis: Book Review. Akash Deep, Sandeep Kumar (Eds.); Elsevier 2019; ISBN: 978-0-12-817456-2

**DOI:** 10.3390/bios9030101

**Published:** 2019-08-22

**Authors:** Ajeet Kaushik

**Affiliations:** Laboratory for Environmental and Biomedical Nanotechnology, Department of Natural Sciences, Division of Sciences, Arts & Mathematics, Florida Polytechnic University, Lakeland, FL 33805-8531, USA; akaushik@floridapoly.edu or

**Keywords:** nanotechnology, nanosensor, diagnostics, environmental monitoring, health wellness, diseases management, point-of-care

## Abstract

A book entitled “Advances in Nanosensors for Biological and Environmental Analysis” published by Elsevier in 2019, is reviewed carefully and critically in this report. In this book, editors explored nanotechnology assisted approaches to develop smart and efficient nanosensors for biological and environmental analysis. Fundamental approaches to prototype development and a focus towards designing miniaturized sensing systems and for point-of-care application, along with considering commercial aspects are key features of this book. This book has potential to serve as a foundation platform for scholars of various disciplines to plan and manage multi-display research in the field of biomedical nanotechnology for diagnostics and environmental monitoring.

## Discussion

For better and accessible health for everybody, the investigation of smart systems is in urgent demand for health and environmental monitoring [1,2,3,4,5,6,7,8,9,10,11,12,13]. To achieve this mission, sensors are emerging as an essential analytical tool for effective diagnostics, therapy efficacy assessment, disease progression monitoring, and targeted biomarker detection for environment quality evaluation. Advancements in sensing component development along with optimized integration made biosensing very smart, which meets patients’ requirements for health and wellness. Further in the interfacing of biosensing prototypes with microfluidic systems and the internet of medical things made biosensing based diagnostics accessible at point-of-care (POC). Further, the introduction of artificial intelligence is also becoming a very important tool to analyze biosensing data to understand and predict trends to correlate prescribed therapies with patient health. To develop a biosensor of desired performance, it is crucial to optimize and develop a biosensing platform which allows and provides for the desired micro-environment to a bio-active needed for maximum binding without compromising the functionality for performing selective sensing. Advancements in nanoscience and nanotechnology, surface-functionalized nano-systems are useful to amplify the signal to achieve higher sensitivity useful to detect a targeted biomarker at low concentrations (pM or fM). The outcomes of these sensing strategies provide useful information needed to optimize effective therapy in a timely manner, understand epidemic variabilities, and design and develop new therapeutics. Keeping advancements, challenges, and prospects of the biosensor in mind, the herein reviewed book “Advances in Nanosensors for Biological and Environmental Analysis” [1] is very well-planned and organized. Each chapter is nicely presented, supported by theory, and includes state-of-art technology, challenges, and prospects. 

The focus of this book is exploring the nano-enabled sensor for biological and environmental applications. The various nano-enabled smart surface-functionalized structure adopted for sensing system development, potential biomarkers and interface of bio-active/nanostructures are covered very well. Additionally, the concept for POC sensing using affordable, and sensitive disposable electrodes made of paper and flexible substrates is a very important part of this book. The challenges associated with developing POC smart sensors for biomedical applications, the potential alternatives to overcoming obstacles, and commercial prospects of state of art nano-sensors are described very well here. 

It has been suggested and verified that a nano-structure of tunable and desired properties plays an important role in developing a sensitive and selective nanosensor, due to enhanced charge transport and maximum immobilization of a bio-active. Chapter 1, (Carbon-based Nanomaterials for the Development of Sensitive Nanosensor) of this book explains the involvement and importance of carbon-based nanomaterials, for example fullerenes, nanotubes, nanodots, and graphene to develop a highly sensitive sensing platform. The strategies describing thin film fabrication, surface functionalization, and stepwise fabrication along with the characterization of a sensor are very well described and illustrated. Various types of sensing applications of carbon-based sensors are presented and well supported.

Chapter 2, (Advances in the Synthesis and Development of Two-Dimensional Transition Metal Dichalcogenides Based Nano-sensor Platforms) starts with the significance of two dimensional (2D) nano-platforms to develop a sensing system. As a focus, Transition Metal Dichalcogenides (TMD) are selected as an example by the authors. The 2D thin film exhibits a large surface area which is useful for the higher loading of bioactive molecules due to intra-/intermolecular interactions. The TMD thin film of MoS_2_, WS_2_, MoSe_2_ exhibit new electrical properties due to easy localization of charge carriers along the *x*-/and *y*-axes. These tunable features of charge localization and managing interlayer spacing presents 2D TMD as potential nanostructures for sensing application. The surface functionality of TMD is a very important factor in immobilized targeted biomolecule such as genes to detect a biomarker at a very low level to evaluate environmental and biological aspects. The advancements in TMD based sensors are illustrated via data and schemes very well in this chapter.

Chapter 3, (Conducting Polymers and Metal-organic Frameworks as Advanced Materials Used for the Development of Nano-sensor Interfaces) describes the significance of conducting polymers for making a sensor of desired performance. It is noticed that conducting polymer are very effective sensing but exhibits a serious problem in its stability. Keeping this in mind, the author highlights metal-organic framework (MOFs) which are a relatively new class of sensing material. The MOFs are known for showing new properties which are absent in their precursors. At the same time, MOFs overcome the challenges exhibited by polymers and inorganic nanoparticles. Due to these salient features and based on organic-inorganic hybrid nanocomposite theory, MOFs are very much suitable nanostructure for sensing fabrication. However, the processing of MOFs needs significant efforts to promote them as a sensing platform. The fundamentals and theories of MOFs describing unique properties needed for the making of a good sensor are described nicely and supported by appropriate illustrations.

Chapter 4, (*Synthesis and Production of Different Biomolecules for Application in the Sensing of Environmental Pollutants*) describes the role of a biorecognition molecule (enzyme, DNA, aptamers, antibody, etc.) to fabricate a nanosensor for selective detection of a targeted marker. As a focus, the author selected biomolecules designed for environmental pollutant detection. This chapter covers various biomolecules and associated optimized synthesis, hypotheses, and rational. Besides, the challenges related to the needs of development, bio-molecules for pollutant selectively, and alternative approaches to overcome the obstacles are also discussed well. However, I do believe that this chapter should be arranged as chapter one, this arrangement would help readers to understand significance of bio-recognition ejectments followed up by their interfacing with nano-structures described in chapters 1 to 3. 

Chapter 5, (*Bioconjugation of Different Nano-surfaces with Biorecognition Molecules for The Development of selective Nano-sensor Platforms*) explores the significance of the optimized binding of a bio-recognition element with a nano-structure. A good binding between them results in (1) amplified signal useful for high selectivity, (2) maximum binding of bioactive molecule useful for wide detection range, and (3) stable sensing systems. State-of-art synthesis, surface functionalization, strategies to enhance optical/electrical/molecular properties of various nanostructures are discussed by authors and well-supported by illustrations and a table. 

Chapter 6, (*Development of Disposable Sensor Strips for Point-of-care Testing of Environmental Pollutants*) starts with very description of the POC testing system significance to achieve rapid detection at a location site. However, the focus of the author is towards environmental monitoring using various detection strategies such as immuno-assays, calorimetric, bio-chemical, electrochemical, and optical analyses, etc. Every strategy is presented and supported by an appropriate illustration. The advantages of a POC system over conventional methodologies, the limitations of state-of-art POC systems, alternative approaches to developing next-generation nano-sensing systems, and prospects towards clinical application are discussed in this chapter nicely and scientifically. 

Chapter 7, (*Advantages and Limitations of Environmental Nano-sensors*) is a very good extension of the previous chapter’s conclusion. Authors describe the advantages of nano-enabled sensing systems over traditional approaches in detail. The miniaturized features of POC sensing and their targeted applications in pollutant detection, soil quality assessment, toxicity evaluation, and analysis, etc., is the strength of this chapter. To achieve sensitive detection, a chip-based sensing approach supported by nanoscience and technology advancements (functionalized smart substrate) is also discussed well in this chapter. Authors also discuss the concerns raised in aspects of sensor processing, packaging, and market strategy towards a translation approach.

Chapter 8, (*Commercial Aspects of Environmental Nano-sensor*) explains the commercial aspects of biosensors developed for diagnostics and environmental monitoring. This chapter covers various aspects of sensor fabrication, optimization, and commercialization. At the same time, attention towards patient requirements, (for example, affordability and accessibility) are very impressive and informative. However, the conclusion of the book along with challenges and future strategies appears missing, but the impact is limited as every chapter is balanced. 

As a final remark, supported by credentials in nanoscience technology accomplishments [2,3,4,5,6,7,8,9,10,11,12,13], Advances in Nanosensors for Biological and Environmental Analysis covers an impressive overview of the field of nanotechnology-based biosensors for diagnostics and environmental monitoring. This book explains the selection and optimization of a bio-recognition element, highly sensitive sensing substrates, surface-functionalized smart nano-systems, sensor fabrication strategies, and commercial aspects. Overall, this is a multidisciplinary book designed for researchers and students who are planning or conducting research in the field of developing a nanotechnology-assisted smart sensors at POC for diagnostics and environmental monitoring. 
